# Incidence of Acute Myocardial Infarction in Hungary: A Nationwide Study

**DOI:** 10.3390/jcm15062318

**Published:** 2026-03-18

**Authors:** Klára Rácz, Gábor Tóth, Elek Dinya, János Németh

**Affiliations:** 1 National Directorate General for Hospitals, Diós Árok 3, H-1125 Budapest, Hungary; racz.klara@okfo.gov.hu; 2 Kispest Health Institute, Ady Endre Út 122, H-1195 Budapest, Hungary; gabortothgabor@gmail.com; 3 Institute of Digital Health Sciences, Semmelweis University, Ferenc tér 15, H-1094 Budapest, Hungary; dinya.elek@semmelweis.hu; 4 Department of Ophthalmology, Semmelweis University, Mária utca 39, H-1085 Budapest, Hungary

**Keywords:** myocardial infarction, ischaemic heart disease, incidence, epidemiology, Hungary

## Abstract

**Background/Objective:** Acute myocardial infarction (AMI) is a common, life-threatening condition and represents a substantial disease burden in Hungary. The aim of this study was to estimate the incidence of AMI in Hungary. **Methods:** This nationwide, retrospective, longitudinal study used data from the National Health Insurance Fund and included patients aged ≥15 years who were newly diagnosed with AMI (ICD-10 codes I21 or I22) between 1 January 2019 and 31 December 2023. Age-standardized incidence rates and their regional distributions were calculated using the European Standard Population from 2013. **Results:** A total of 16,171 and 14,797 patients with AMI were identified in 2019 and 2023, respectively, showing a declining trend (−1.60%; 95% CI: −2.10% to −1.10%; *p* < 0.0001). Age-standardized incidence rates varied between 144.22 and 166.63/100,000 person-years (PYs) during the analyzed period. The highest age-standardized incidence was detected among men (235.75/100,000 PYs) in 2019. The annual decrease in AMI incidence was significantly greater (*p* = 0.003) among women (−2.60%; 95% CI: −3.39% to −1.80%) than among men (−1.06%; 95% CI: −1.71% to −0.41%). **Conclusions:** The incidence of AMI in Hungary was in line with findings from other studies conducted in Central and Eastern European countries. AMI incidence showed a decreasing trend during the analyzed period. Men had higher incidence rates, and the declining trend was more pronounced among women.

## 1. Introduction

Ischemic heart disease (IHD) is a leading cause of mortality worldwide, accounting for 9.1 million deaths in 2019 [1].

Although substantial advances have been made in both preventive strategies and therapeutic interventions, acute myocardial infarction (AMI)—a severe manifestation of IHD—remains a common, life-threatening condition, accounting for approximately three quarters of all IHD deaths, and continues to place a considerable burden on healthcare systems in higher-income countries, including Hungary [2,3].

Hypertension, hypertriglyceridemia, obesity, diabetes, sedentary lifestyle, and tobacco use are recognized as the principal risk factors. Arterial stiffness, a major risk factor for AMI, is more severe in patients with metabolic syndrome [4]. Moreover, men, people of older age, and those with lower socioeconomic status are disproportionately affected [5].

The vulnerable population is steadily increasing worldwide due to population aging and the growing prevalence of obesity and diabetes [6]. Nevertheless, improvements in both primary and secondary prevention may effectively reduce the economic burden associated with IHD [7]. In recent years, the incidence and mortality of AMI have declined in Europe and the United States [8]. Several studies have examined the epidemiology of AMI in Europe and globally [9,10,11]. However, no recent population-based studies reporting AMI incidence rates, specifically in Hungary, are scarce across Europe [12,13]. Given the substantial public health burden of IHD, monitoring trends in AMI incidence is essential.

As a nationwide population-based database and the country’s sole health insurance provider, the National Health Insurance Fund’s (NHIF) database offers the most accurate source of information regarding AMI incidence in Hungary. Epidemiological research based on the NHIF database has been shown to be a reliable and valid method in recent years [14,15]. The aim of our study was to estimate the incidence of AMI and its regional distribution among people aged 15 years and older in Hungary.

## 2. Materials and Methods

### 2.1. Study Design

We used data from the NHIF, the primary public healthcare financing authority in Hungary. The NHIF database covers nearly the entire Hungarian population and contains information on patient demographics and medical diagnoses coded according to the International Classification of Diseases, 10 (ICD-10). In Hungary, the NHIF fully finances all acute medical care and interventions related to AMI, as no alternative insurance system provides coverage for AMI treatment.

Our nationwide, retrospective, longitudinal study included all acutely admitted AMI patients aged ≥ 15 years at the time of diagnosis, with a main diagnostic code of ICD-10 I21 or I22, who were diagnosed and treated as inpatients between 1 January 2019 and 31 December 2023. Hospitalizations occurring within 40 days were considered a single case. Data were anonymized during data collection, and only non-identifiable data were processed for analysis.

The AMI cases and incidence rates were represented as crude numbers and age-standardized rates. Mid-year population size data for Hungary given by age and sex for standardization were obtained from the Hungarian Central Statistical Office (HCSO). Age-standardized rates [per 100,000 person-years (PYs)] were calculated from crude incidence numbers using the 2013 European Standard Population (ESP) to facilitate comparisons with earlier studies. We analyzed the total and annual changes in incidence of AMI in Hungary.

All incidence rates were calculated for the seven main regions of Hungary (Central Hungary, Northern Great Plain, Southern Great Plain, Northern Hungary, Central Transdanubia, Southern Transdanubia, and Western Transdanubia). For regional comparisons of AMI incidence, Central Hungary served as the reference region because it is the most developed region in Hungary, has the largest population, and includes the capital city, Budapest.

### 2.2. Statistical Analysis

Changes in age-standardized incidence rates of AMI over time were analyzed using Poisson regression models with a log link. Annual mean changes in the incidence rates were determined using regression models with 95% confidence intervals (CIs). Between 2019 and 2023, the number of AMI events was considered the outcome variable, and the logarithm of the mid-year population size was included as an offset term in the regression model to account for differences in population size across data, thereby modeling incidence rates rather than raw counts. Calendar year served as the explanatory variable. To examine potential geographical differences, region was included as a categorical predictor using reference-cell coding with Central Hungary as the reference category. Incidence rate ratios (IRR) and their corresponding 95% CIs were obtained by exponentiating the regression coefficients and their Wald-based confidence limits. Model assumptions were evaluated by examining goodness-of-fit statistics. Overdispersion in the Poisson models was assessed using the ratio of the deviance and Pearson χ^2^ statistics to their respective degrees of freedom. Sensitivity analyses were performed to ensure that the main findings were robust to potential deviations from model assumptions. Where evidence of extra-Poisson variation was detected, dispersion-adjusted standard errors were used to ensure robust inference. In addition, to formally evaluate whether temporal trends differed between men and women, models including a sex × calendar year interaction term were fitted, and the statistical significance of the interaction was assessed using Wald tests.

We also calculated the incidence rates with the corresponding 95% CI values for each year, and comparisons between genders were also performed. The CI values were calculated according to the recommended method provided by Altman et al. [16]. The incidence and population data of the NHIF in Hungary were standardized using HCSO data for the examined period (2019–2023). Age standardization calculations were based on the typical methodological foundations published by Jensen et al. and dos Santos Silva [17,18].

Statistical significance was set at *p* < 0.05. All calculations were performed using SAS software (version 9.4, TS1M9, 2025 by SAS Institute Inc., Cary, NC, USA).

## 3. Results

### 3.1. Crude Numbers

Crude incidence and crude regional incidences are shown in Table S1. Totally, 73,836 people were newly diagnosed with AMI between 2019 and 2023, 60.6% of whom were men. We found 16,171 and 14,797 new AMI cases in 2019 and 2023, respectively, corresponding to 0.19% and 0.17% of the entire Hungarian population at risk. Ratio of men with AMI fluctuated from 59.5% to 61.6% during 2019–2023. The incidence of AMI increased with age. AMI occurred most frequently among people between 60 and 69 years (Table 1).

### 3.2. Incidence

**Figure 1:**
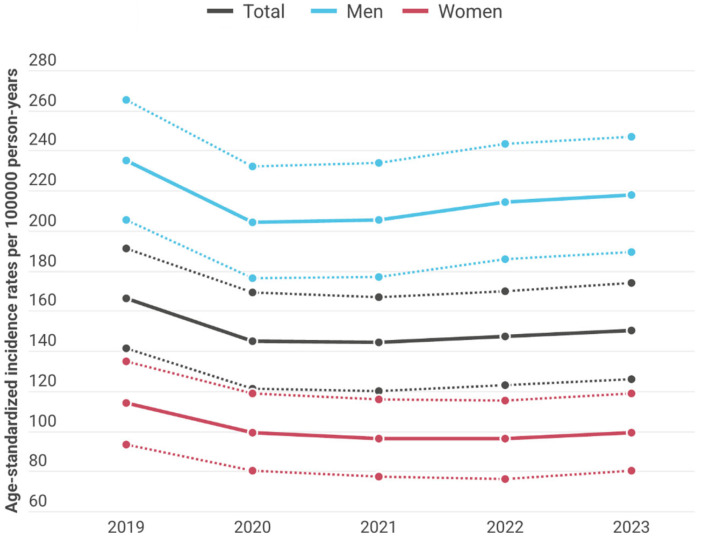
Age-standardized incidence rates (European Standard Population 2013) of acute myocardial infarction by gender in Hungary between 2019 and 2023 (per 100,000 person-years; dotted lines represent 95% CI).

Age-standardized incidence fluctuated between 144.22/100,000 PYs (95% CI: 120.68–167.76) and 166.63/100,000 PYs (95% CI: 141.33–191.93) during 2019–2023 in the total study population of Hungary (Figure 1). Age-standardized incidence of AMI changed by −1.60% annually (95% CI: −2.10% to −1.10%; *p* < 0.0001) in the entire study population, by −1.06% (95% CI: −1.71% to −0.41%; *p* = 0.0015) in men and by −2.60% (95% CI: −3.39% to −1.80%; *p* < 0.0001) in women. The annual decrease in the incidence of AMI was significantly larger among women than among men (*p* = 0.003).

Age-standardized incidence rates were higher among men than in women through all analyzed years with the lowest rate in 2020 (204.78/100,000 PYs; 95% CI: 176.72–232.84) and the highest in 2019 (235.75/100,000 PYs; 95% CI: 205.65–265.85).

### 3.3. Regional Incidence

**Figure 2:**
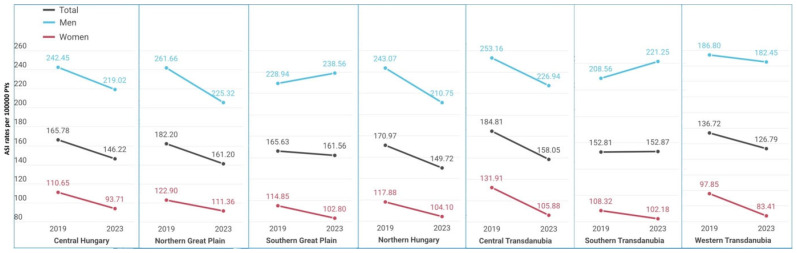
Age-standardized incidence rates (European Standard Population 2013) of acute myocardial infarction per 100,000 person-years in the main regions of Hungary in 2019 and 2023.

The highest age-standardized incidence rates in the entire population were recorded in Central Transdanubia in 2019 (184.81/100,000 PYs) and in Southern Great Plain in 2023 (161.56/100,000 PYs). Age-standardized incidence rates showed a decreasing trend in almost every region between 2019 and 2023 (Figure 2). The lowest incidence rates in the total population were found in Western Transdanubia both in 2019 and 2023 (136.72/100,000 PYs and 126.79/100,000 PYs, respectively). The greatest and significant change in incidence was observed in Northern Hungary (−2.70%; 95% CI: −4.14% to −1.25%; *p* = 0.0003) (Table 2).

The highest age-standardized incidence rates in men were recorded in the Northern Great Plain in 2019 (261.66/100,000 PYs) and in the Southern Great Plain in 2023 (238.56/100,000 PYs). The lowest incidence rates among men were found in Western Transdanubia both in 2019 and 2023 (186.80/100,000 PYs and 182.45/100,000 PYs, respectively) (Figure 2). AMI incidence change showed a declining trend among men in Northern Hungary (−2.97%; 95% CI: −4.82% to −1.08%; *p* = 0.002) and in Northern Great Plain (−1.87%; 95% CI: −3.48% to −0.23%; *p* = 0.026). The highest AMI incidence in women was registered in Central Transdanubia (131.91/100,000 PYs in 2019 and 105.88/100,000 PYs in 2023), and the lowest incidence was in the Western Transdanubian region (97.85/100,000 PYs in 2019 and 77.69/100,000 PYs in 2022). The greatest and significant changes in incidence were observed in Central Transdanubia (−4.23%; 95% CI: −6.46% to −1.96%); *p* = 0.0003) and in Western Transdanubia (−4.17%; 95% CI: −6.74% to −1.54%; *p* = 0.002).

The relative rate of AMI was evaluated in the study year 2023 using the Central Hungarian region as a reference (Table 3). Compared with the Central Hungary reference region, we found a significantly higher rate of AMI across the entire country (IRR ≥ 1.08; *p* ≤ 0.0048) except in Western Transdanubia (0.93; 95% CI: 0.87–0.99), where the incidence of AMI was significantly lower (*p* = 0.017) than in Central Hungary.

## 4. Discussion

As far as we know, this Hungarian study is the first nationwide study on incidence of AMI and its regional distribution. Epidemiological aspects of AMI are important because AMI is one of the leading causes of mortality [8]. The NHIF database is the most authoritative source of health care data in Hungary, and our study provides detailed information on the incidence of AMI for the entire Hungarian population over recent years.

The incidence rates of AMI showed a decreasing trend over the 5-year study period, ranging from 144.22 to 166.63 cases per 100,000 between 2019 and 2023 in the total population of Hungary, and the incidence increased with age.

Main risk factors of AMI are uncontrolled high blood pressure, smoking, hypercholesterinemia and sedentary lifestyle [2]. Smoking has lost popularity in recent years [7]. The prevalence of smoking decreased from 34% in 2003 to 28% in 2014 Hungary but has not declined significantly since then [19,20]. However, due to the ban on smoking in public spaces, the prevalence of secondhand smoke exposure has decreased since [21]. The prevalence of uncontrolled high blood pressure and hypercholesterinemia is also showing a decreasing trend [22,23]. In addition, lower target levels for low-density lipoprotein cholesterol and blood pressure may result in better control of cardiovascular risk factors [24]. Other explanations for the decreasing incidence may include improvements in primary prevention and cardiological care across the country, as well as better tertiary prevention through the use of antiplatelet therapy [2]. In addition, the increasing prevalence of major risk factors such as obesity and diabetes, as well as population aging, may have an opposing effect [24,25]. Regarding structural characteristics, a concave-shaped chest wall has been reported to be associated with a lower incidence of AMI [26], whereas mitral valve prolapse does not appear to increase the risk of AMI [27]. Recent research from North America and Europe indicates that AMI incidence, which remained relatively stable during the 1990 s, began to decline after 2000 [28].

Incidence rates and trends of AMI vary between countries due to the different risk factors, population structures, study designs and observed age groups. The decrease in the incidence of AMI in Hungary was consistent with reports from other European countries [13]. A decreasing trend in AMI incidence was reported in the USA after 2000 [29], in Sweden between 2001 and 2008 [30], in the Netherlands from 1998 to 2007 [31], and in England from 2002 to 2010 [3]. In Poland [32], the geographically closest country, the incidence of AMI reported in 2012 was higher (235/100,000 PYs) than the incidence observed in Hungary between 2019 and 2023 (144.22–166.63/100,000 PYs). Consistent with our data, AMI incidence decreased from 473.2/100,000 PYs in 1994 to 192.7/100,000 PYs in 2016 in the Czech Republic [33]. Earlier data from Hungary also suggest a decreasing trend in AMI incidence, as Jánosi et al.’s [34] registry study, which had incomplete population coverage and a narrower observed age group (population aged ≥ 30 years), reported an incidence of 177.5/100,000 PYs in 2010–2013. The observed variability in AMI incidence across European countries is likely multifactorial. These differences may arise from variations in population age structure, as well as differences in the prevalence of major cardiovascular risk factors, lifestyle patterns, socioeconomic conditions, and access to preventive healthcare [8]. In addition, differences in healthcare systems, diagnostic practices, reporting methods, and the quality and completeness of national registries may also contribute to the variability observed between countries [35,36]. The decline observed in 2020 and 2021 may be partly explained by changes in reporting practices, as NHIF-funded healthcare providers in Hungary temporarily shifted from fee-for-service reimbursement to average-based financing during the COVID-19 pandemic. In addition, stringent governmental restrictions and nationwide lockdown measures were implemented in spring 2020 to mitigate viral transmission. Together, reduced healthcare utilization and limited access to medical services during this period may have contributed to the lower recorded incidence of AMI in 2020–2021 [15,37] and may have led to an underestimation of the true incidence of AMI. However, as the present study did not directly analyze the impact of the COVID-19 pandemic, these explanations should be interpreted with caution.

Our study found a higher incidence of AMI in men and elderly people, consistent with international data, and a significantly greater decline in incidence among women. The higher incidence in men may be partly explained by a higher prevalence of smoking among men compared with women (31.1% vs. 25.4% in 2019) [20] and poorer health-seeking behaviors among men [38]. However, over the past decades, the lifestyles and risk behaviors of men and women have become more similar [2]. Nevertheless, risk factors may have different effects in the two genders. Women experience more stress than men, and mental stress can more than double the risk of AMI [39,40]. Women with diabetes have a 40% higher risk of AMI than men [41]. Gestational diabetes, hypertension during pregnancy, polycystic ovary syndrome, and menopause-related changes are risk factors specific to women. Women have poorer control of blood pressure, blood sugar, and lipid levels than men, even when receiving the same treatment, and women who smoke have a higher risk of IHD [42]. In contrast, studies have shown that, at the same levels of blood pressure and cholesterol, men tend to develop more severe disease [43]. Men are more likely to develop IHD at a younger age than women [41]. The prevalence of diabetes and hypertension is higher in younger men, but this trend changes after 60 years of age, when blood pressure and diabetes prevalence become higher among women [12,25]. Given the aging population in Hungary and the longer life expectancy of women, the prevalence of IHD is increasing among women, because sex hormones lessen the impact of cardiovascular risk factors during the reproductive years [44]. In addition, the implementation of primary prevention appears to be more effective in women than in men [7]. With respect to secondary and tertiary prevention, several studies have reported that female gender and older age are the main risk factors for medication non-adherence [42]. However, a meta-analysis showed that, for antihypertensive therapy, there is no overall gender difference in medication adherence, except among patients older than 65 years, in whom adherence is better in men [45]. The more pronounced decline in women may be partly attributable to increased awareness of IHD among women and to a reduction in gender differences [9].

In line with nationwide data, incidence of AMI in men was higher compared to women in all regions. Our study demonstrated significant geographic disparities in AMI incidence. The most pronounced declining trends were detected in the two least developed regions (Northern Hungary and the Northern Great Plain) as well as in the most developed region (Central Hungary). In addition, in direct regional comparisons, AMI incidence was lowest in the two most developed regions (Central Hungary and Western Transdanubia). In 2023, gross domestic product per capita was highest in Central Hungary at more than EUR 33,000, followed by Western Transdanubia at EUR 17,800, while it was EUR 12,700 in Northern Hungary and EUR 13,100 in the Northern Great Plain [46]. Differences in regional AMI incidence may be related to the more favorable socioeconomic conditions and better access to primary health care services in more developed regions [47]. These patterns are consistent with previous research suggesting that regional differences in AMI incidence may be related to variation in socioeconomic conditions, lifestyle factors, preventive health care programs, and access to primary care. The incidence of AMI is strongly associated with socioeconomic factors, including educational level, occupation, income, and social environment, as well as with unhealthy diet and a higher prevalence of smoking, diabetes, and uncontrolled hypertension [48,49]. However, as the present study did not directly include socioeconomic indicators, comorbidity profiles, or measures of health care access, these explanations should be interpreted with caution.

The declining trend in AMI incidence could be further strengthened and regional inequalities reduced through optimized preventive strategies. Nationwide primary prevention remains essential. Health awareness should be promoted through health education within the public education system, alongside policies that support healthier lifestyles, such as reducing taxes on basic healthy foods and improving their availability in public catering. Stronger tobacco control measures and improved access to opportunities for physical activity could further contribute to lowering AMI incidence. In addition, strengthening primary care, improving access to dietetic services, and increasing reimbursement levels for preventive medications could enhance secondary and tertiary prevention. These measures may contribute to further reductions in AMI incidence and improve long-term quality of life among affected individuals [50,51].

Limitations of this study include that undiagnosed (limited information on out-of-hospital deaths) and unmanaged AMI cases were not included in our sample, which may cause a slight underestimation of the incidence of AMI. Although nationwide estimates are lacking, international studies indicate that approximately 20% of myocardial infarctions are undiagnosed [52]. The NHIF database is an administrative database with manually coded entries for billing purposes; therefore, both undercoding and overcoding are possible. However, coding inaccuracies are less likely for frequently occurring diagnoses for which hospitals have financial incentives to code accurately, such as AMI [3]. Nevertheless, the diagnoses could not be further verified. Only main diagnostic codes were screened; therefore, patients with AMI listed as an additional diagnostic code may have been missed, which could also lead to a small underestimation of the incidence. Our study lacked detailed clinical and patient-level information. Because only recurrent AMI occurring within the first 40 days could be excluded, recurrent events beyond 40 days may have led to a slight overestimation of the incidence, although the proportion of recurrent AMI among all AMI cases is estimated to be 1.9–2.9% [53]. Distinguishing between ST-elevation and non-ST-elevation AMI was not feasible due to limitations of the NHIF database.

Strengths of our study include the large sample size, complete nationwide coverage and standardized methodology used in Hungary.

## 5. Conclusions

In conclusion, the incidence of AMI in Hungary fluctuated between 144.22 and 166.63 per 100,000 PYs. AMI incidence showed a decreasing trend between 2019 and 2023, in line with findings from other studies conducted in Central and Eastern European countries. Men had higher incidence rates, while the declining trend was more pronounced among women. Our study contributes to a better understanding of the epidemiology of AMI in Central and Eastern Europe. By addressing a gap in the epidemiological data on AMI in Hungary, this study facilitates comparisons across countries and regions and provides valuable baseline data for the analysis of future trends. Further examination of epidemiological aspects of AMI, such as mortality and its regional characteristics, could provide a more detailed view of the potentially uneven geographical distribution of deficiencies and weaknesses in the Hungarian healthcare system and help identify the most important areas for improvement.

## Figures and Tables

**Table 1:** Aggregated age and gender composition of people with new acute myocardial infarction in Hungary between 2019 and 2023.

Age Groups (Years)	Men		Women		Total	
	N	%	N	%	N	%
15–19	12	0.02	1	0.003	13	0.02
20–29	103	0.2	33	0.1	136	0.2
30–39	769	1.7	261	0.9	1030	1.4
40–49	4778	10.6	1515	5.2	6293	8.5
50–59	9434	21.1	3543	12.2	12,977	17.6
60–69	13,512	30.2	7292	25.0	20,804	28.1
70–79	11,122	24.9	8738	30.0	19,860	26.9
80+	4994	11.2	7729	26.5	12,723	17.2
Total	44,724	100	29,112	100	73,836	100

**Table 2:** Annual change in age-standardized incidence rates (European Standard Population 2013) of acute myocardial infarction per 100,000 person-years in Hungary and its main regions between 2019 and 2023.

	Men [Change (%); *p*]	Women [Change (%); *p*]	Total [Change (%); *p*]
Central Hungary	−1.11 (−2.31 to 0.11); 0.074	−3.08 (−4.54 to −1.59); <0.001	−1.86 (−2.79 to −0.92); 0.0001
Central Transdanubia	−0.86 (−2.72 to 1.03); 0.37	−4.23 (−6.46 to −1.96); 0.0003	−2.21 (−3.64 to −0.75); 0.003
Western Transdanubia	0.58 (−1.58 to 2.79); 0.60	−4.17 (−6.74 to −1.54); 0.002	−1.31 (−2.96 to 0.38); 0.13
Southern Transdanubia	1.71 (−0.48 to 3.95); 0.13	−1.97 (−4.52 to 0.64); 0.14	0.21 (−1.46 to 1.90); 0.80
Northern Hungary	−2.97 (−4.82 to −1.08); 0.002	−2.41 (−4.65 to −0.12); 0.039	−2.70 (−4.14 to −1.25); 0.0003
Northern Great Plain	−1.87 (−3.48 to −0.23); 0.026	−1.55 (−3.60 to 0.55); 0.14	−1.73 (−3.00 to −0.44); 0.008
Southern Great Plain	0.72 (−1.05 to 2.52); 0.43	0.19 (−1.96 to 2.37); 0.87	0.74 (−0.63 to 2.13); 0.29

**Table 3:** Incidence rate ratios of acute myocardial infarction across Hungary’s regions in 2023, with the Central Hungarian region as the reference, in the total population (**A**), men (**B**), and women (**C**). Significant *p* values are bold. CI = confidence interval.

(A) Total	New Cases	Incidence Rate Ratio	95% CI	*p*
Central Hungary	4289	1 (reference)		
Central Transdanubia	1733	1.14	1.08–1.21	**<0.001**
Western Transdanubia	1311	0.93	0.87–0.99	**0.017**
Southern Transdanubia	1453	2.22	2.10–2.36	**<0.001**
Northern Hungary	1677	1.08	1.03–1.15	**0.0048**
Northern Great Plain	2238	1.13	1.07–1.19	**<0.001**
Southern Great Plain	2096	1.22	1.15–1.28	**<0.001**
**(B) Men**				
Central Hungary	2636	1 (reference)		
Central Transdanubia	1063	1.11	1.03–1.19	**0.0037**
Western Transdanubia	812	0.91	0.85–0.99	**0.026**
Southern Transdanubia	889	1.16	1.07–1.25	**<0.001**
Northern Hungary	993	1.03	0.96–1.11	0.45
Northern Great Plain	1348	1.08	1.01–1.16	**0.018**
Southern Great Plain	1305	1.21	1.14–1.30	**<0.001**
**(C) Women**				
Central Hungary	1653	1 (reference)		
Central Transdanubia	670	1.17	1.07–1.29	**<0.001**
Western Transdanubia	499	0.93	0.85–1.03	0.18
Southern Transdanubia	564	1.19	1.08–1.31	**<0.001**
Northern Hungary	684	1.16	1.07–1.27	**<0.001**
Northern Great Plain	890	1.19	1.10–1.29	**<0.001**
Southern Great Plain	791	1.21	1.11–1.31	**<0.001**

## Data Availability

The raw data supporting the conclusions of this article will be made available by the authors upon request.

## Supplementary Materials

The following supporting information can be downloaded at: https://www.mdpi.com/article/10.3390/jcm15062318/s1, Table S1. Crude numbers and incidences, of acute myocardial infarction in Hungary between 2019 and 2023.

## Abbreviations

The following abbreviations are used in this manuscript:

AMI	acute myocardial infarction
CI	confidence interval
ESP	European Standard Population
ICD	International Classification of Diseases
IHD	ischemic heart disease
IRR	incidence rate ratio
HCSO	Hungarian Central Statistical Office
NHIF	National Health Insurance Fund
PYs	person-years
