# Supplementary material for: Incidence of Acute Myocardial Infarction in Hungary: A Nationwide Study
Source: J Clin Med. 2026 Mar 18;15(6):2318. doi: 10.3390/jcm15062318 (PMC13026180; doi:10.3390/jcm15062318)
Supplement: Supplementary file 1 [file jcm-15-02318-s001.zip › jcm-4168418-supplementary.pdf]

**Table S1.** Crude numbers and incidences, of acute myocardial infarction in Hungary between 2019 and 2023.

| **Incidence** | **Number of patients** | | | | | | | | | |
|---|---|---|---|---|---|---|---|---|---|---|
| | **2019** | | **2020** | | **2021** | | **2022** | | **2023** | |
| | **N** | **%** | **N** | **%** | **N** | **%** | **N** | **%** | **N** | **%** |
| **Total country (n)** | 16171 | 0.19 | 14249 | 0.17 | 14182 | 0.17 | 14437 | 0.17 | 14797 | 0.17 |
| Men | 9628 | 59.5 | 8542 | 59.9 | 8607 | 60.7 | 8901 | 61.6 | 9046 | 61.1 |
| Women | 6543 | 40.5 | 5707 | 40.1 | 5575 | 39.3 | 5536 | 38.4 | 5751 | 38.9 |
| **Regions (n)** | | | | | | | | | | |
| *Central Hungary* | *4800* | *0.19* | *3909* | *0.16* | *3974* | *0.16* | *4130* | *0.17* | *4289* | *0.17* |
| Men | 2882 | 60.0 | 2348 | 60.0 | 2409 | 61.0 | 2570 | 62.2 | 2636 | 61.4 |
| Women | 1918 | 40.0 | 1561 | 40.0 | 1565 | 39.0 | 1560 | 37.8 | 1653 | 38.6 |
| *Central Transdanubia* | *1965* | *0.21* | *1702* | *0.18* | *1715* | *0.18* | *1748* | *0.19* | *1733* | *0.19* |
| Men | 1152 | 58.6 | 1023 | 60.1 | 1036 | 60.4 | 1094 | 62.6 | 1063 | 61.3 |
| Women | 813 | 41.4 | 679 | 39.9 | 679 | 39.6 | 654 | 37.4 | 670 | 38.7 |
| *Western Transdanubia* | *1361* | *0.16* | *1346* | *0.15* | *1389* | *0.16* | *1292* | *0.15* | *1311* | *0.15* |
| Men | 795 | 58.4 | 802 | 59.6 | 844 | 60.8 | 828 | 64.1 | 812 | 61.9 |
| Women | 566 | 41.6 | 544 | 40.4 | 545 | 39.2 | 464 | 35.9 | 499 | 38.1 |
| Southern Transdanubia | *1443* | *0.18* | *1370* | *0.18* | *1263* | *0.16* | *1300* | *0.17* | *1453* | *0.19* |
| Men | 847 | 58.7 | 778 | 56.8 | 759 | 60.1 | 788 | 60.6 | 889 | 61.2 |
| Women | 596 | 41.3 | 592 | 43.2 | 504 | 39.9 | 512 | 39.4 | 564 | 38.8 |
| *Northern Hungary* | *1942* | *0.20* | *1780* | *0.18* | *1643* | *0.17* | *1700* | *0.18* | *1677* | *0.17* |
| Men | 1154 | 59.4 | 1047 | 58.8 | 985 | 59.9 | 998 | 58.7 | 993 | 59.2 |
| Women | 788 | 40.6 | 733 | 41.2 | 658 | 40.1 | 702 | 41.3 | 684 | 40.8 |
| *Northern Great Plain* | *2514* | *0.20* | *2216* | *0.18* | *2150* | *0.17* | *2225* | *0.18* | *2238* | *0.18* |
| Men | 1539 | 61.2 | 1378 | 62.2 | 1335 | 62.1 | 1414 | 63.5 | 1348 | 60.2 |
| Women | 975 | 38.8 | 838 | 37.8 | 815 | 37.9 | 811 | 36.5 | 890 | 39.8 |
| *Southern Great Plain* | *2146* | *0.20* | *1926* | *0.18* | *2048* | *0.19* | *2042* | *0.19* | *2096* | *0.20* |
| Men | 1259 | 58.7 | 1166 | 60.5 | 1239 | 60.5 | 1209 | 59.2 | 1305 | 62.3 |
| Women | 887 | 41.3 | 760 | 39.5 | 809 | 39.5 | 833 | 40.8 | 791 | 37.7 |
